# Structural basis of AlpA-dependent transcription antitermination

**DOI:** 10.1093/nar/gkac608

**Published:** 2022-07-25

**Authors:** Aijia Wen, Minxing Zhao, Sha Jin, Yuan-Qiang Lu, Yu Feng

**Affiliations:** Department of Biophysics, and Department of Infectious Disease of Sir Run Run Shaw Hospital, Zhejiang University School of Medicine, Hangzhou 310058, China; Department of Emergency Medicine of the First Affiliated Hospital, Zhejiang University School of Medicine, Hangzhou 310003, China; Department of Biophysics, and Department of Infectious Disease of Sir Run Run Shaw Hospital, Zhejiang University School of Medicine, Hangzhou 310058, China; Department of Emergency Medicine of the First Affiliated Hospital, Zhejiang University School of Medicine, Hangzhou 310003, China; Department of Biophysics, and Department of Infectious Disease of Sir Run Run Shaw Hospital, Zhejiang University School of Medicine, Hangzhou 310058, China; Zhejiang Provincial Key Laboratory of Immunity and Inflammatory diseases, Hangzhou 310058, China

## Abstract

AlpA positively regulates a programmed cell death pathway linked to the virulence of *Pseudomonas aeruginosa* by recognizing an AlpA binding element within the promoter, then binding RNA polymerase directly and allowing it to bypass an intrinsic terminator positioned downstream. Here, we report the single-particle cryo-electron microscopy structures of both an AlpA-loading complex and an AlpA-loaded complex. These structures indicate that the C-terminal helix-turn-helix motif of AlpA binds to the AlpA binding element and that the N-terminal segment of AlpA forms a narrow ring inside the RNA exit channel. AlpA was also revealed to render RNAP resistant to termination signals by prohibiting RNA hairpin formation in the RNA exit channel. Structural analysis predicted that AlpA, 21Q, λQ and 82Q share the same mechanism of transcription antitermination.

## INTRODUCTION

**Figure 1.**
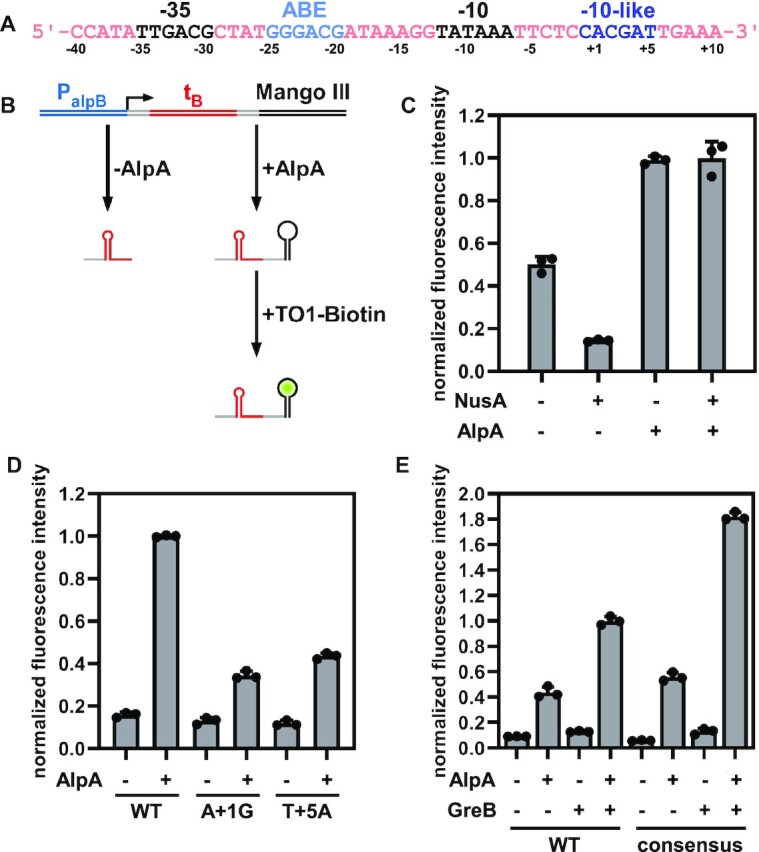
A σ-dependent pause is required for AlpA-dependent transcription antitermination. (**A**) Sequence of the *alpB* promoter. Black, −35 and −10 elements; light blue, AlpA binding element; dark blue, −10-like sequence. Positions are numbered relative to the transcription start site. (**B**) Principle of the Mango III transcription assay. (**C**) t_B_ is a weak terminator and requires NusA to be fully active. *In vitro* transcription assay was performed in the presence of GreB and components identified below each bar. In order to normalize the data, a DNA template lacking the terminator sequence was constructed and *in vitro* transcription assay was performed with GreB alone. Then the reading is used to normalize the graph globally. (**D**) −10-like sequence is required for AlpA-dependent transcription antitermination. *In vitro* transcription assay was performed in the presence of GreB, NusA, and components identified below each bar. The fluorescence intensity is normalized as in (C). (**E**) GreB enhances AlpA-dependent transcription antitermination. *In vitro* transcription assay was performed in the presence of NusA and components identified below each bar. The fluorescence intensity is normalized as in (C).

*Pseudomonas aeruginosa* (*Pae*) is an opportunistic pathogen affecting immunocompromised patients. It is the leading cause of morbidity and mortality in cystic fibrosis patients and one of the leading causes of nosocomial infections (1–3). Due to a range of mechanisms for adaptation, survival, and resistance to multiple classes of antibiotics, infection by *Pae* can be life-threatening and is emerging worldwide as a public health threat. The programmed cell death (PCD) pathway encoded by the genes *alpRABCDE* contributes to the virulence of *Pae* (4). AlpR is a repressor that undergoes auto-cleavage in response to DNA damage resulting in derepression of *alpA*. AlpA then positively regulates the cell lysis genes *alpBCDE* by functioning as a processive antiterminator (5). In particular, AlpA recognizes a putative AlpA binding element (ABE) located between the −10 and −35 elements of the *alpB* promoter (P*_alpB_*, Figure 1A), binds RNAP directly, and allows it to bypass an intrinsic termination site (t_B_) positioned downstream of P*_alpB_*.

Bacteriophage Q proteins have served as a paradigm for studying transcription antitermination for more than 40 years (6–9). All Q proteins require two cis-acting elements embedded within phage late gene promoters to engage RNA polymerase (RNAP): a −10-like sequence and a Q binding element (QBE) (10). The −10-like sequence is located in the initially transcribed region, resembles the promoter −10 element, and causes a σ-dependent pause (11–13). Q proteins engage the paused RNAP when bound to the QBE, forming a Q-loading complex. The Q-loading complex then escapes the pause and transforms into a Q-loaded complex, which is resistant to termination signals. Failure to escape the σ-dependent pause results in backtracking, and GreB rescues backtracked RNAP by cutting off the 3′ end of backtracked RNA (14,15). The elongation factor NusA may enhance the antitermination activity of Q proteins, as well. Biochemical experiments have demonstrated that NusA is required in order for Q protein originating from bacteriophage 82 to construct a stable complex with RNAP (16).

In previous work, we determined a single-particle cryo-electron microscopy (cryo-EM) structure of the Q-loading complex from bacteriophage 21 (17). The structure shows that two 21Q protomers (Q^I^ and Q^II^) bind to the QBE simultaneously and contact distinct elements of the RNA exit channel. Notably, Q^I^ forms a narrow ring inside the RNA exit channel. A cryo-EM structure of the 21Q-loaded complex has also been determined, revealing only one 21Q protomer, corresponding to Q^I^, that interacts with the RNA exit channel and has the RNA 5′ end threaded through its ring (18). Both structures suggest that 21Q acts as a molecular nozzle to prevent the formation of terminator RNA hairpins.

In this study, we verify that a σ-dependent pause is required for AlpA-dependent transcription antitermination. We determined the cryo-EM structures of an AlpA-loading complex and an AlpA-loaded complex. Unlike 21Q, only one AlpA molecule binds to the ABE and RNA exit channel in the loading complex. Like 21Q, AlpA forms a narrow ring inside the RNA exit channel and renders RNAP resistant to termination signals by prohibiting RNA hairpin formation.

## MATERIALS AND METHODS

### Protein expression and purification

AlpA and AlpA derivatives were prepared from *Escherichia coli* strain BL21(DE3) (Invitrogen, Inc.) transformed with plasmid pET21a-*alpA* encoding AlpA (NCBI accession number: WP_034080184) under the control of T7 promoter. Single colonies were used to inoculate 1 L LB broth containing 100 μg/ml ampicillin, cultures were incubated at 37°C with shaking until OD_600_ = 0.6, cultures were induced by addition of IPTG to 1 mM, and cultures were incubated 18 h at 16°C. Then cells were harvested by centrifugation (5,000 x g; 10 min at 4°C), resuspended in 20 ml buffer A (10 mM Tris–HCl, pH 7.5, 0.2 M NaCl, 5% glycerol, 1 mM EDTA, and 1 mM DTT) and lysed using a JN-02C cell disrupter (JNBIO, Inc.). The lysate was centrifuged (20 000 × g; 45 min at 4°C), and the supernatant was loaded onto a 5 ml column of HiTrap Heparin HP (GE Healthcare, Inc.) equilibrated in buffer A and eluted with a 100 ml linear gradient of 0.2–1 M NaCl in buffer A. The sample was further purified by cation-exchange chromatography on a Mono S 10/100 GL column (GE Healthcare, Inc.; 160 ml linear gradient of 0.1–1 M NaCl in buffer A). Fractions containing AlpA were pooled and stored at −80°C. Yields were ∼0.5–2 mg/l, and purities were >95%.

*Pae* RNAP core enzyme was prepared from *E. coli* strain BL21(DE3) (Invitrogen, Inc.) transformed with plasmids pCOLA-*Pae rpoB-rpoC* and pACYC-*Pae rpoA-rpoZ*. Single colonies of the resulting transformants were used to inoculate 100 ml LB broth containing 50 μg/ml kanamycin and 34 μg/ml chloramphenicol, and cultures were incubated 16 h at 37°C with shaking. Aliquots (5 ml) were used to inoculate 1 L LB broth containing 50 μg/ml kanamycin and 34 μg/ml chloramphenicol, cultures were incubated at 37°C with shaking until OD_600_ = 0.6, cultures were induced by addition of IPTG to 0.5 mM, and cultures were incubated 18 h at 18°C. Then cells were harvested by centrifugation (5000 × g; 10 min at 4°C), resuspended in 20 ml lysis buffer (40 mM Tris–HCl, pH 8.0, 0.2 M NaCl, 2 mM EDTA, 5% glycerol and 2 mM DTT) and lysed using a JN-02C cell disrupter (JNBIO, Inc.). After poly(ethyleneimine) precipitation and ammonium sulfate precipitation, the pellet was resuspended in buffer B (10 mM Tris–HCl, pH 8.0, 0.4 M NaCl, 5% glycerol and 1 mM DTT) and loaded onto a 6 ml column of Ni-NTA 6FF agarose (Smart-Lifesciences, Inc.) equilibrated with buffer B. The column was washed with 30 ml buffer B containing 20 mM imidazole and eluted with 30 ml buffer B containing 0.16 M imidazole. The eluate was diluted and loaded onto a HiTrap Q HP column (GE Healthcare, Inc.) equilibrated in buffer C (10 mM Tris–HCl, pH8.0, 0.2 M NaCl, 1 mM EDTA, 1 mM DTT and 5% glycerol) and eluted with a 160 ml linear gradient of 0.2–0.6 M NaCl in buffer C. Fractions containing *Pae* RNAP core enzyme were pooled and stored at −80°C. Yield was ∼3.5 mg/l, and purity was > 95%.

*Pae* σ^70^ was prepared from *E. coli* strain BL21(DE3) (Invitrogen, Inc.) transformed with plasmid pET26b-*Pae* σ^70^ encoding C-hexahistidine-tagged *Pae* σ^70^ under the control of T7 promoter. Single colonies of the resulting transformants were used to inoculate 50 ml LB broth containing 50 μg/ml kanamycin, and cultures were incubated 16 h at 37°C with shaking. Aliquots (10 ml) were used to inoculate 1 l LB broth containing 50 μg/ml kanamycin, cultures were incubated at 37°C with shaking until OD_600_ = 0.6, cultures were induced by addition of IPTG to 1 mM, and cultures were incubated an additional 14 h at 18°C. Cells were harvested by centrifugation (5000 × g; 10 min at 4°C), resuspended in 20 ml buffer D (50 mM Tris–HCl, pH 7.5, 0.2 M NaCl, 1 mM DTT) and lysed using a JN-02C cell disrupter (JNBIO, Inc.). The lysate was centrifuged (20 000 × g; 45 min at 4°C), and the supernatant was loaded onto a 2 ml column of Ni-NTA 6FF agarose (Smart-Lifesciences, Inc.) equilibrated with buffer D. The column was washed with 10 ml buffer D containing 20 mM imidazole and eluted with 10 ml buffer D containing 0.16 M imidazole. The sample was further purified by anion-exchange chromatography on a HiTrap Q HP column (GE Healthcare, Inc.; 160 ml linear gradient of 0.1–1 M NaCl in buffer D). The fractions were concentrated using an Amicon Ultra-15 centrifugal filter (10 kDa MWCO; Merck Millipore, Inc.) and applied to a HiLoad 16/600 Superdex 200 column (GE Healthcare, Inc.) equilibrated in 10 mM HEPES, pH 7.5, and 50 mM KCl. The column was eluted with 120 ml of the same buffer. Fractions containing *Pae* σ^70^ were pooled and stored at −80°C. Yields were ∼9 mg/l, and purities were >95%.

*Pae* RNAP holoenzyme was prepared by incubating *Pae* RNAP core enzyme and *Pae* σ^70^ in a 1:3 ratio for 1 h at 4°C. The reaction mixture was applied to a HiLoad 16/600 Superdex 200 column (GE Healthcare, Inc.) equilibrated in 10 mM HEPES, pH 7.5 and 50 mM KCl, and the column was eluted with 120 ml of the same buffer. Fractions containing *Pae* RNAP-σ^70^ holoenzyme were pooled and stored at −80°C. GreB and NusA were purified as reported (19,20).

### Mango III transcription antitermination assay

A DNA fragment corresponding to −154 to + 247 of the *Pae alpB* promoter followed by Mango III coding sequence was synthesized and inserted into pUC57 (GENEWIZ, Inc.). The DNA fragment was amplified by PCR, was purified using the FastPure Gel DNA Extraction Mini Kit (Vazyme, Inc.), and was stored at −80°C. *Pae alpB* promoter derivatives were prepared using site-directed mutagenesis. Transcription antitermination assay was performed in a 384-well microplate format. Reaction mixtures contained (50 μl): 0 or 0.1 μM AlpA, 0 or 0.1 μM GreB, 0 or 0.2 μM NusA, 0.1 μM *Pae* RNAP-σ^70^ holoenzyme, 20 nM DNA fragment, 1 μM TO1-Biotin, 0.2 mM ATP, 0.2 mM UTP, 0.2 mM GTP, 0.2 mM CTP, 50 mM Tris–HCl, pH 8.0, 0.1 M KCl, 10 mM MgCl_2_, 1 mM DTT and 5% glycerol. Following incubation for 15 min at 37°C, fluorescence emission intensities were measured using an Infinite M200 Pro microplate reader (TECAN, Inc.; excitation wavelength = 500 nm; emission wavelength = 540 nm).

### Assembly and structural determination of AlpA-loading complex

Template strand DNA, nontemplate strand DNA and RNA were annealed at a 1:1:1 ratio in 50 mM Tris–HCl, pH 8.0, 0.1 M KCl and stored at −80°C. Reaction mixture contained (31 μl): 32 μM AlpA, 32 μM *Pae* RNAP core, 32 μM *Pae* σ^70^, 32 μM DNA scaffold, 50 mM Tris–HCl, pH 8.0, 0.1 M KCl, 10 mM MgCl_2_, 1 mM DTT. Reaction mixture was incubated 10 min at room temperature. Immediately before freezing, 8 mM CHAPSO was added to the sample. Quantifoil grids (R 1.2/1.3, Cu, 300) were glow-discharged for 120 s at 25 mA prior to the application of 3 μl of the complex, then plunge-frozen in liquid ethane using a Vitrobot (FEI, Inc.) with 95% chamber humidity at 10°C.

The grids were imaged using a 300 kV Titan Krios equipped with a Falcon 4 direct electron detector (FEI, Inc.). Images were recorded with EPU in counting mode with a physical pixel size of 0.93 Å and a defocus range of 1.0–2.0 μm. Images were recorded with a 7 s exposure to give a total dose of 62 e/Å^2^. Subframes were aligned and summed using RELION’s own implementation of the UCSF MotionCor2 (21). The contrast transfer function was estimated for each summed image using CTFFIND4 (22). From the summed images, ∼10 000 particles were manually picked and subjected to 2D classification in RELION (23). 2D averages of the best classes were used as templates for auto-picking in RELION. After 2D classification, particles were 3D classified in RELION using a map of 21Q-loading complex (EMD-9852) (17) low-pass filtered to 40 Å resolution as a reference. 3D classification resulted in 4 classes, among which only one class has a clear density for RNAP. Particles in this class were 3D auto-refined, then subjected to 3D classification focused on the RNA exit channel without alignment. Focused 3D classification resulted in 4 classes, among which class 2 has a clear density for AlpA, as expected for AlpA-loading complex, while class 4 has a clear density for σR4 as in RNAP-promoter open complex. From this classification, particles in class 2 was 3D auto-refined and post-processed in RELION.

The models of RNAP, σR2 and nucleic acids from the structure of 21Q-loading complex (PDB 6JNX) (17), and the model of AlpA predicted by AlphaFold (24) were fitted into the cryo-EM density map using Chimera (25) and were adjusted in Coot (26). The coordinates were real-space refined with secondary structure restraints in Phenix (27).

### Assembly and structural determination of AlpA-loaded complex

A DNA fragment corresponding to *Pae alpB* promoter followed by a 68-bp C-less cassette containing a consensus −10-like sequence, followed by a CC-halt site was synthesized and inserted into pUC57 (GENEWIZ, Inc.). The DNA fragment was amplified by PCR, was purified using the FastPure Gel DNA Extraction Mini Kit (Vazyme, Inc.), and was stored at −80°C. Reaction mixture contained (2 ml): 2 μM AlpA, 1 μM *Pae* RNAP-σ^70^ holoenzyme, 0.8 μM DNA scaffold, 1 μM GreB, 1 mM ATP, 1 mM GTP, 1 mM UTP, 50 mM Tris–HCl, pH 8.0, 0.1 M KCl, 10 mM MgCl_2_, 1 mM DTT. Reaction mixture was incubated 10 min at 37°C. Reaction mixture was concentrated to 50 μl using an Amicon Ultra-0.5ml centrifugal filter (100 kDa MWCO; Merck Millipore, Inc.). Immediately before freezing, 8 mM CHAPSO was added to the sample. Quantifoil grids (R 1.2/1.3, Cu, 300) were glow-discharged for 120 s at 25 mA prior to the application of 3 μl of the complex, then plunge-frozen in liquid ethane using a Vitrobot (FEI, Inc.) with 95% chamber humidity at 4°C.

The grids were imaged using a 300 kV Titan Krios equipped with a Falcon 4 direct electron detector (FEI, Inc.). Images were recorded with EPU in counting mode with a physical pixel size of 0.93 Å and a defocus range of 0.9–1.8 μm. Images were recorded with a 6 s exposure to give a total dose of 52 e/Å^2^. Subframes were aligned and summed using RELION’s own implementation of the UCSF MotionCor2 (21). The contrast transfer function was estimated for each summed image using CTFFIND4 (22). From the summed images, approximately 10,000 particles were manually picked and subjected to 2D classification in RELION (23). 2D averages of the best classes were used as templates for auto-picking in RELION. After 2D classification, particles were 3D classified in RELION using a map of AlpA-loading complex low-pass filtered to 40 Å resolution as a reference. 3D classification resulted in 4 classes, among which only one class has a clear density for RNAP. Further 3D classification results in two classes with clear density for σ^70^ and two classes without density for σ^70^. Particles without density for σ^70^ were combined, then subjected to 3D classification focused on the RNA exit channel without alignment. Focused 3D classification resulted in 4 classes, among which class 2 has a clear density for AlpA, as expected for AlpA-loaded complex. From this classification, particles in class 2 was 3D auto-refined and post-processed in RELION.

The models of RNAP and AlpA from the structure of AlpA-loading complex, and the model of nucleic acids from the structure of 21Q-loaded complex (PDB 6P19) were fitted into the cryo-EM density map using Chimera (25) and were adjusted in Coot (26). The coordinates were real-space refined with secondary structure restraints in Phenix (27).

## RESULTS

### A σ-dependent pause is required for AlpA-dependent transcription antitermination

To ascertain the minimal requirements for AlpA-dependent transcription antitermination, we purified recombinant *Pae* RNAP, σ^70^ and AlpA, and developed an *in vitro* transcription assay by taking advantage of an RNA fluorogenic aptamer, Mango III (Figure 1B and Supplementary Figure S1). Specifically, a DNA fragment consisting of the promoter P*_alpB_* and terminator t_B_ followed by a Mango III encoding sequence is transcribed *in vitro*. If RNAP reads through terminator t_B_, the Mango III encoding sequence is transcribed, and the transcript becomes fluorescent when bound to TO1-Biotin.

NusA can enhance termination at weak intrinsic terminators by stimulating hairpin formation and stabilizing the hairpin's interaction with the flap (28–31). However, NusA can also facilitate the antitermination of N protein from bacteriophage λ and P7 protein from bacteriophage Xp10 by stabilizing the antiterminator-RNAP complexes (32–35). First, we tested whether NusA enhances termination at t_B_ in the absence of AlpA and found that the fluorescence intensity with NusA is significantly lower than without NusA (Figure 1C), indicating that t_B_ is a weak terminator and needs NusA to be fully active. We then tested whether NusA facilitates AlpA-dependent transcription antitermination, finding that fluorescence intensity is not increased by NusA, which indicates that NusA doesn’t enhance the antitermination activity of AlpA.

Since σ-dependent pauses provide the time window for Q proteins to engage RNAP, we wondered whether a σ-dependent pause is also required for AlpA-dependent transcription antitermination. By inspecting promoter P*_alpB_*, we found a potential −10-like sequence (5′-CACGAT-3′) positioned from −1 to + 5, which could be recognized by σ^70^ and mediate a σ-dependent pause. When we substituted the consensus A at position + 1 or the consensus T at position + 5 of the potential −10-like sequence, the antitermination activity of AlpA was jeopardized (Figure 1D), verifying that a σ-dependent pause is required for AlpA-dependent transcription antitermination.

Failure to escape the σ-dependent pause leads to backtracking, which can be rescued by GreB. To test whether RNAP backtracks on P*_alpB_*, *in vitro* transcription assays were performed with and without GreB (Figure 1E). The results show that GreB enhances fluorescence intensities in the presence and absence of AlpA, indicating that RNAP backtracks on P*_alpB_* regardless of the presence or absence of AlpA. The effect of GreB is more dramatic when the −10-like sequence is changed to the consensus sequence (5′-TATAAT-3′), consistent with previous reports that the strength of the interaction between σR2 and the −10-like sequence can profoundly impact the probability of backtracking (12,13).

### Cryo-EM structure of AlpA-loading complex

**Figure 2.**
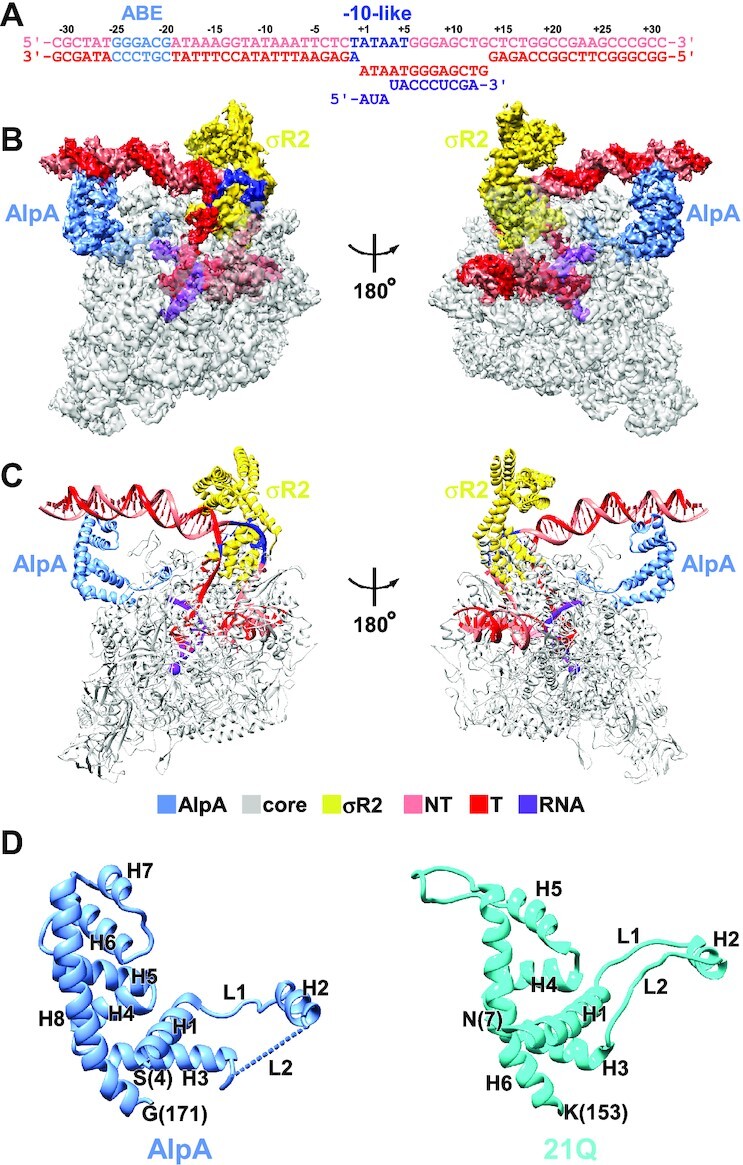
Cryo-EM structure of AlpA-loading complex. (**A**) Nucleic-acid scaffold sequence used for cryo-EM. Salmon, nontemplate strand; red, template strand; purple, RNA; light blue, ABE; dark blue, −10-like sequence. Positions are numbered relative to the transcription start site. (**B, C**) The cryo-EM density map without B-factor sharpening (B) and the model (C) of the AlpA-loading complex. Gray, RNAP; light blue, AlpA; yellow, σR2; purple, active center Mg^2+^ and RNA; salmon, nontemplate strand; red, template strand; dark blue, −10-like sequence. (**D**) AlpA is structurally similar to 21Q (PDB 6JNX).

To obtain the structure of an AlpA-loading complex, we used a nucleic-acid scaffold corresponding to positions −31 to + 31 of promoter P*_alpB_* (Figure 2A). The scaffold contains an ABE, a consensus −10-like sequence, a 13-bp transcription bubble maintained in the unwound state by having non-complementary sequences on nontemplate and template strands, and a 12-nt RNA. We incubated the scaffold with *Pae* RNAP, σ^70^, and AlpA, froze the sample, and collected data using Titan Krios. 3D classification revealed the structure of the AlpA-loading complex, determined at a nominal resolution of 3.3 Å (Figure 2B, C, Supplementary Figures S2-S4, and Table S1). A local resolution calculation indicated that the central core of the structure is determined to a 3.0–4.0 Å resolution (Supplementary Figure S3C).

The experimental density map shows unambiguous densities for RNAP, σR2, AlpA and the nucleic-acid scaffold (Figure 2B and Supplementary Figure S4). The RNAP and σR2 of the structure are very similar to the cryo-EM structure of the 21Q-loading complex (17), with a root-mean-square deviation (RMSD) of 0.917 Å (3455 Cαs aligned). In addition, σR2 binds to the −10-like sequence in the same way as in 21Q-loading complex and RNAP-promoter open complex (36–40), in accordance with the observation that the −10-like sequence is critical for AlpA-dependent transcription antitermination. σ^70^ conserved regions σR3, σR3.2 and σR4 are displaced from RNAP, probably due to the steric clashes between σR3.2, σR4, and the 5′ end of nascent RNA, consistent with the fact that σR3, σR3.2 and σR4 are dispensable for σ-dependent pause (41).

### AlpA is structurally similar to 21Q

In the cryo-EM density map, there is one density feature adjacent to the RNA exit channel, which can be attributed to AlpA. The structural model of AlpA predicted by AlphaFold (24) can be fit into the density feature with only a minor adjustment (Supplementary Figure S4B). AlpA is composed of eight helices (H1–H8) and two loops (L1 and L2), among which the density map for L1, the loop connecting H2 and H3, is too weak to build a reliable model (Figure 2D). The N-terminal segment of AlpA, including L1, L2 and H2 forms a ring-like structure, while the C-terminal segment of AlpA, including H7 and H8, forms a helix-turn-helix motif. Although AlpA bears no sequence homology to 21Q, the folding of AlpA is strikingly reminiscent of 21Q, except that two more helices (H5 and H6) are inserted in AlpA (Figure 2D), hinting that AlpA exerts its antitermination activity through an analogous mechanism.

### AlpA–ABE interactions that mediate AlpA engagement

**Figure 3.**
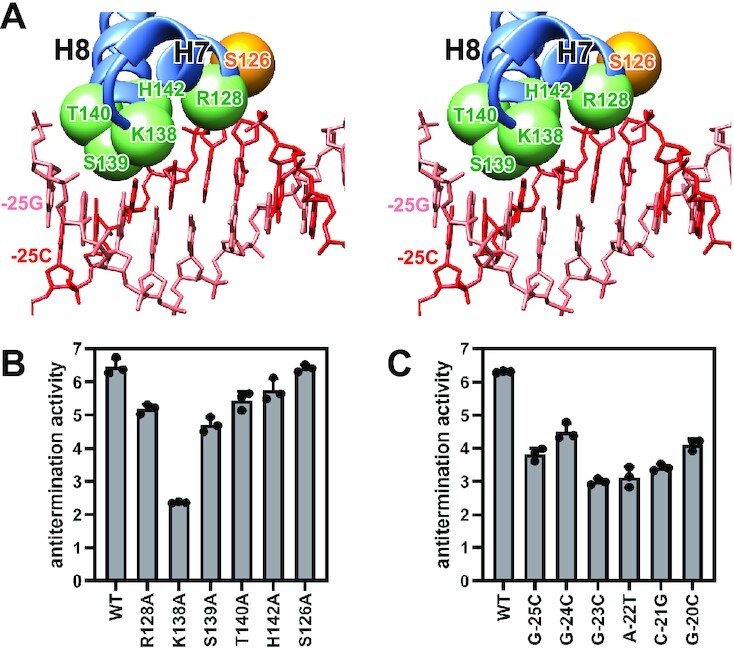
AlpA-ABE interactions that mediate AlpA engagement. (**A**) Interactions between AlpA and ABE are depicted in stereo view. Salmon, nontemplate strand; red, template strand; light blue, AlpA. The Cα atoms of potential contact residues are shown as green spheres. The Cα atom of AlpA residue S126 is shown as an orange sphere. (**B**) Effects on antitermination of alanine substitution of potential contact residues. S126A serves as a negative control. Antitermination activity is calculated by dividing the fluorescence intensity in the presence of AlpA by the fluorescence intensity in the absence of AlpA. Two-tailed, unpaired, unequal variance *t*-tests are used to calculate *p*-values between indicated samples. WT versus R128A, *P* = 0.0034; WT versus K138A, *P* = 0.0012; WT versus S139A, *P* = 0.0009; WT versus T140A, *P* = 0.0092; WT versus H142A, *P* = 0.0460; WT versus S126A, *P* = 0.7867. (**C**) Effects on antitermination of substitution of each base pair of ABE. Antitermination activity is calculated by dividing the fluorescence intensity in the presence of AlpA by the fluorescence intensity in the absence of AlpA. Two-tailed, unpaired, unequal variance *t*-tests are used to calculate *P*-values between indicated samples. WT versus G–25C, *P* = 0.0016; WT versus G–24C, *P* = 0.0060; WT versus G–23C, *P* = 0.0001; WT versus A–22T, *P* = 0.0030; WT versus C–21G, *P* = 0.0002; WT versus G–20C, *P* = 0.0018.

In the AlpA-loading complex structure, the helix-turn-helix motif formed by H7 and H8 participates in the recognition of ABE (Figure 3A, Supplementary Figure S4C). Specifically, residues R128, K138, S139, T140 and H142 are positioned in the major groove, potentially making interactions with DNA that could enable sequence readout. Consistent with the AlpA-loading complex structure, alanine substitution of these residues, but not the nearby residue S126, reduces AlpA-dependent read-through, verifying their functional importance (Figure 3A and B). Furthermore, the substitution of every base pair of ABE (5′-GGGACG-3′) affects AlpA-dependent transcription antitermination (Figure 3C), indicating that every base pair of ABE is essential for AlpA-dependent transcription antitermination.

### AlpA-RNAP interactions that mediate AlpA engagement

**Figure 4.**
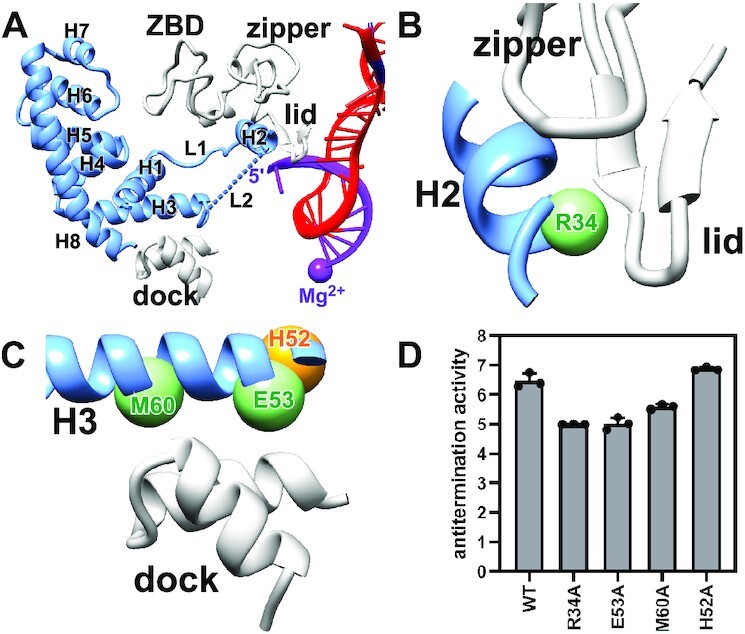
AlpA-RNAP interactions that mediate AlpA engagement. (**A**) H3 sits on the exterior opening of the RNA exit channel and interacts with the dock, while H2 inserts into the RNA exit channel and interacts with the zipper, lid, and zinc binding domain (ZBD). Gray, RNAP; light blue, AlpA; purple, active center Mg^2+^ and RNA; red, template strand. (**B**) Interaction of H2 with the zipper and lid. The Cα atom of AlpA residue R34 is shown as a sphere. (**C**) Interaction of H3 with the dock. The Cα atoms of AlpA residues E53 and M60 are shown as green spheres. The Cα atom of AlpA residue H52 is shown as an orange sphere. (**D**) Effects on antitermination of alanine substitution of potential contact residues. H52A serves as a negative control. Antitermination activity is calculated by dividing the fluorescence intensity in the presence of AlpA by the fluorescence intensity in the absence of AlpA. Two-tailed, unpaired, unequal variance *t*-tests are used to calculate *P*-values between indicated samples. WT versus R34A, *P* = 0.0093; WT versus E53A, *P* = 0.0017; WT versus M60A, *P* = 0.0186; WT versus H52A, *P* = 0.0931.

Our structure of the AlpA-loading complex reveals that the N-terminal segment of AlpA forms a ring-like structure and contacts various elements of the RNA exit channel (Figure 4A). In particular, H3 sits on the exterior opening of the RNA exit channel and interacts with the dock, while H2 inserts into the RNA exit channel and interacts with the zipper, lid, and zinc binding domain (ZBD). Specifically, H2 residue R34 is positioned to potentially make van der Waals interactions with the zipper and lid (Figure 4B and Supplementary Figure S4D), while H3 residues E53 and M60 are positioned to potentially make electrostatic interactions and van der Waals interactions with the dock (Figure 4C and Supplementary Figure S4E). Alanine substitution of the inferred interacting residues, but not the nearby residue H52, decreases AlpA-dependent antitermination (Figure 4C and D), confirming that the cryo-EM structure is biologically relevant.

### Cryo-EM structure of AlpA-loaded complex

**Figure 5.**
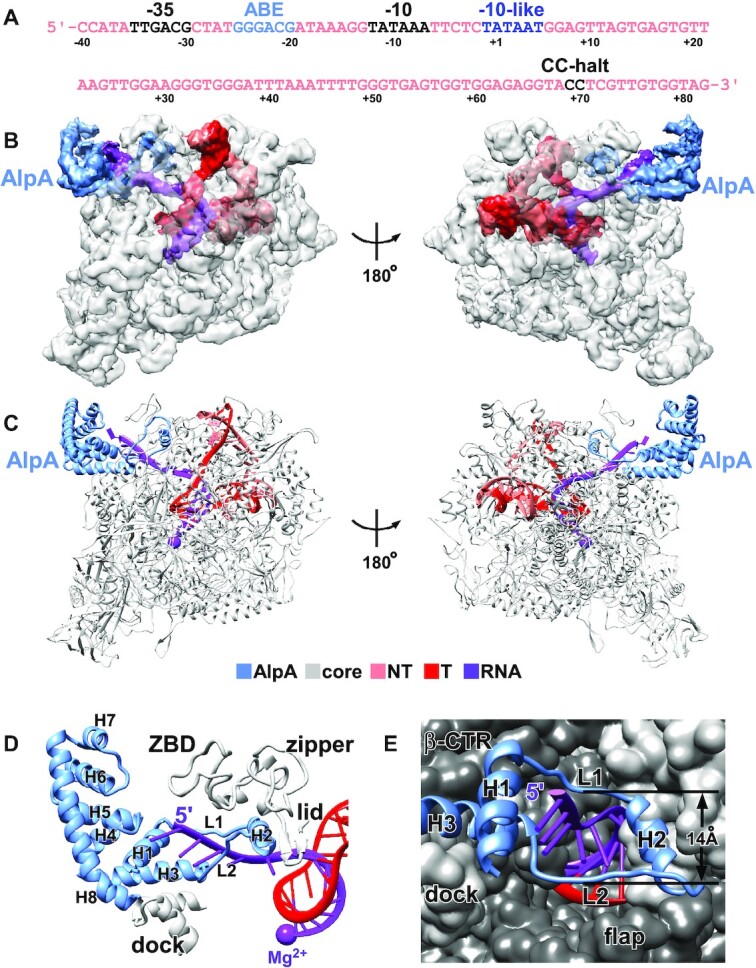
Cryo-EM structure of AlpA-loaded complex. (**A**) The nucleic-acid scaffold sequence used for cryo-EM. Black, CC-halt site, −35 and −10 elements; light blue, ABE; dark blue, −10-like sequence. Positions are numbered relative to the transcription start site. (**B, C**) The cryo-EM density map without B-factor sharpening (B) and the model (C) of the AlpA-loaded complex. Gray, RNAP; light blue, AlpA; purple, active center Mg^2+^ and RNA; salmon, nontemplate strand; red, template strand. (**D**) AlpA interacts with the RNA exit channel and has the RNA 5′ end threaded into and through its ring. Gray, RNAP; light blue, AlpA; purple, active center Mg^2+^ and RNA; red, template strand. (**E**) L1, L2 and H2 form a ring-like structure inside the RNA exit channel. RNAP is shown as surface. The ZBD is omitted for clarity.

To prepare an AlpA-loaded complex, we performed *in vitro* transcription with a DNA template comprising promoter P*_alpB_*, followed by a 68-bp C-less cassette containing a consensus −10-like sequence, followed by a CC-halt site (Figure 5A), using *Pae* RNAP-σ^70^ holoenzyme, AlpA, and GreB, in the presence of an NTP subset lacking CTP. 3D classification revealed three major classes of molecular assemblies: AlpA-loaded complexes (identifiable by the presence of AlpA and absence of σ^70^), transcription elongation complexes (identifiable by the absence of AlpA and σ^70^), and initial transcribing complexes (identifiable by the absence of AlpA and presence of σ^70^, Figure 5B, C, Supplementary Figures S5–S7, and Table S1). The structure of the AlpA-loaded complex was determined at a nominal resolution of 3.7 Å (Supplementary Figure S6A). A local resolution calculation indicated that the central core of the structure is determined to 3.5–4.5 Å resolution (Supplementary Figure S6C). The experimental density map shows unambiguous densities for RNAP, AlpA, and the nucleic-acid scaffold (Figure 5B and Supplementary Figure S7). The RNAP of the structure is very similar to the structure of the AlpA-loading complex, with a root-mean-square deviation (RMSD) of 0.539 Å (3177 Cαs aligned). Extensive 3D classification did not reveal a complex with both AlpA and σ^70^, indicating that σ^70^ is released upon the formation of a loaded complex (Supplementary Figure S5).

The structure of the AlpA-loaded complex reveals AlpA interacting with the RNA exit channel and having the RNA 5′ end threaded into and through its ring (Figure 5B-E). The conformation and interactions of AlpA in the loaded complex are similar to those in the loading complex. In particular, the AlpA N-terminal segment adopts a similar conformation and makes similar interactions with the RNA exit channel (Figure 5D, Supplementary Figure S7C–E). Notable changes include a rotation of the AlpA C-terminal segment as a rigid body (Supplementary Figure S8) associated with the loss of AlpA-DNA interactions in the loaded complex, and a disorder-to-order transition in L1 (Supplementary Figures S4B, S7B) associated with the gain of AlpA-RNA interactions in the loaded complex. Clear, traceable density is present for the 14 nucleotides (nt) at the 3′ end of the RNA product: 2 nt of RNA are located upstream of the AlpA ring, outside the RNA exit channel; 3 nt of RNA are located downstream of the AlpA ring, in the RNA exit channel; and 9 nt of RNA are base paired with the template strand DNA as an RNA–DNA hybrid (Figure 5D, Supplementary Figure S7C).

## DISCUSSION

**Figure 6.**
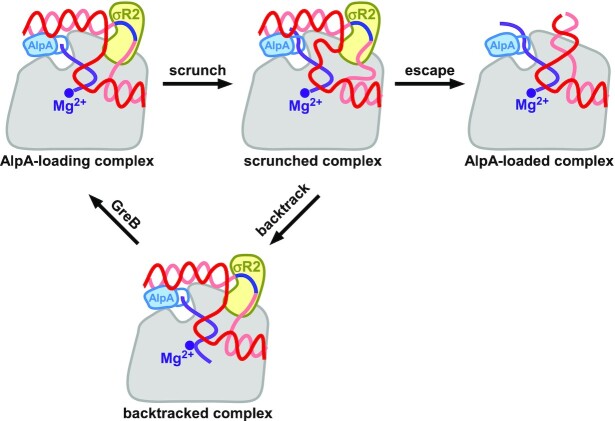
The pathway for the AlpA-loaded complex formation. Gray, RNAP; light blue, AlpA; yellow, σR2; purple, active center Mg^2+^ and RNA; salmon, nontemplate strand; red, template strand; dark blue, −10-like sequence.

A pathway for the formation of the AlpA-loaded complex can be drawn based on our structural and biochemical analyses (Figure 6). During transcription initiation, σR4 and σR2 are anchored to RNAP and make sequence-specific contacts with the promoter −35 element and −10 element, respectively (36–40,42–44). After promoter escape, σR3, σR3.2 and σR4 are displaced because of the steric clashes with the RNA 5′ end, while σR2 is retained (45–47). If a −10-like sequence is encountered, σR2 will make exactly the same contacts with it as in an RNAP-promoter open complex, which will lead to a σ-dependent pause (11–13,41). The interaction between the upstream fork junction of the transcription bubble and σR2 restrains the conformation of upstream dsDNA so that ABE is located in the vicinity of the RNA exit channel. Taking advantage of the pause, AlpA binds to ABE and interacts with the RNA exit channel. Since the −10-like sequence of the transcription bubble is anchored to RNAP through σR2, further extension of the nascent RNA leads to scrunching as in initial transcription (48,49). If the energy stored in the scrunch is insufficient to break the anchoring, RNAP backtracks into an arrested state, and cleavage of the backtracked RNA must occur in order to resume transcription (50). If the energy stored in the scrunch is sufficient to break the anchoring, RNAP escapes from the arrested state and transforms into an AlpA-loaded complex.

The structure of the AlpA-loaded complex reveals how AlpA renders RNAP resistant to intrinsic terminators, which are transcribed to form a GC-rich hairpin followed by a 7–8 nt U-tract (51). According to the structure of the AlpA-loaded complex, the ring-like structure of AlpA inserts into the RNA exit channel and serves as a molecular nozzle (Figure 5D and E). The inner diameter of the nozzle is <14 Å (the shortest distance between Cαs), which is too small to accommodate an RNA hairpin (diameter > 20 Å) (31,52). Therefore, we infer that AlpA renders RNAP resistant to intrinsic termination by prohibition RNA hairpin formation in the RNA exit channel.

The pathway for the formation of the 21Q-loaded complex is more complicated than that of AlpA. The 21Q-loading complex contains two 21Q protomers (Q^I^ and Q^II^, Supplementary Figure S9). Q^I^ makes interactions analogous to those made by AlpA, interacting with the dock and forming a ring-like structure that enters the RNA exit channel. Q^II^ makes interactions absent in the AlpA-loading complex, interacting with the flap tip helix. However, Q^II^ is released during the process of transforming from the loading complex to the loaded complex.

Bacteriophage Q proteins can be divided into three protein families: the 21Q family, the λQ family and the 82Q family (18). Q proteins from the three protein families perform equivalent regulatory functions, but surprisingly, these Q proteins exhibit no significant sequence similarity to each other (6,7,53,54). There is no experimental structure for full-length λQ and 82Q. Although the crystal structure of a λQ fragment has been reported (55), the N-terminal segment, which is critical for λQ-dependent transcription antitermination, is missing in the fragment. To explore the mechanism of λQ and 82Q-dependent transcription antitermination, we predicted their structures using AlphaFold (Supplementary Figure S10). The N-terminal segments of λQ and 82Q form ring-like structures, while the C-terminal segments of λQ and 82Q form helix-turn-helix motifs. While λQ, 82Q and AlpA don’t appear to exhibit any sequence homology, λQ and 82Q adopt similar conformations as AlpA except that a zinc finger is inserted in λQ, hinting that they function through similar mechanisms. Namely, the helix-turn-helix and zinc finger motifs participate in DNA recognition, while the N-terminal segments act as molecular nozzles to prevent the formation of terminator RNA hairpins.

Despite having great variety in their protein sequences, 21Q, λQ, 82Q and AlpA likely share similar tertiary structures and antitermination mechanisms. With the improvement of structure prediction algorithms and implementation of structure-based protein function annotation, more Q-like antiterminators may be found in the vast genome database.

## DATA AVAILABILITY

The accession numbers for the cryo-EM density maps reported in this paper are Electron Microscopy Data Bank: EMD-33515 and EMD-33516. The accession numbers for the atomic coordinates reported in this paper are Protein Data Bank: 7XYA and 7XYB.

## Supplementary Material

gkac608_Supplemental_File
