# Supplementary material for: Structural basis of AlpA-dependent transcription antitermination
Source: Nucleic Acids Res. 2022 Jul 25;50(14):8321–30. doi: 10.1093/nar/gkac608 (PMC9371919; doi:10.1093/nar/gkac608)
Supplement: gkac608_Supplemental_File [file gkac608_supplemental_file.pdf]

Structural basis of AlpA-dependent transcription antitermination

Wen et al.

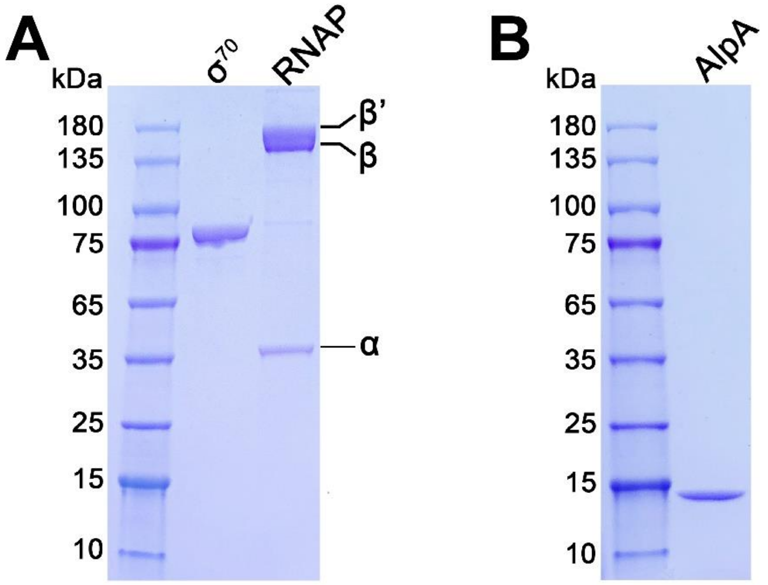

Figure S1. The SDS-PAGE of *Pae* RNAP, σ^{70} (A) and AlpA (B).

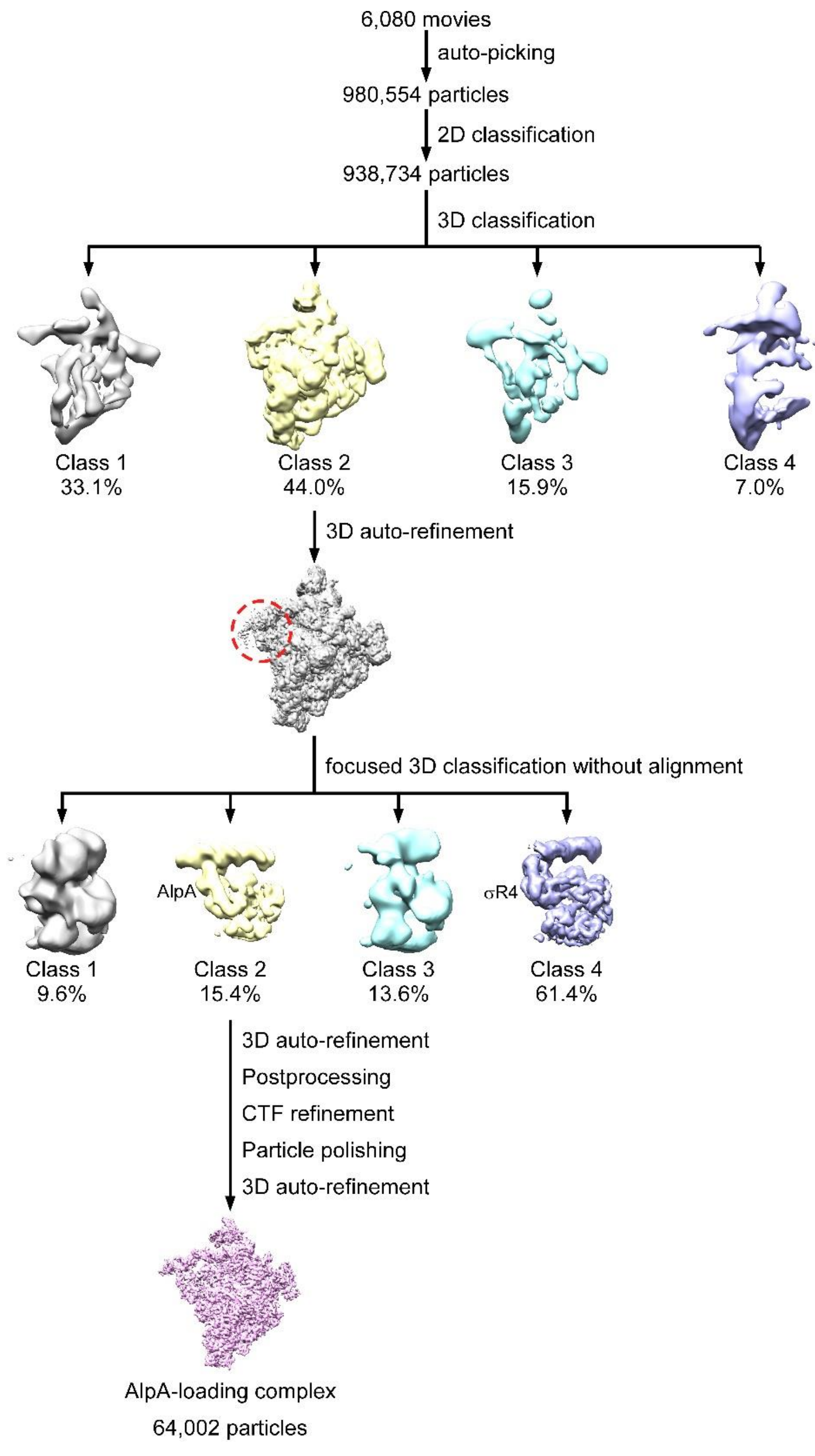

Figure S2. The data processing pipeline for the AlpA-loading complex.

3D classification resulted in 4 classes, among which only one class has a clear density for RNAP. Particles in this class were 3D auto-refined, then subjected to 3D classification focused on the RNA exit channel without alignment. Focused 3D classification resulted in 4 classes, among which class 2 has a clear density for AlpA, as expected for AlpA-loading complex, while class 4 has a clear density for σR4 as in RNAP-promoter open complex.

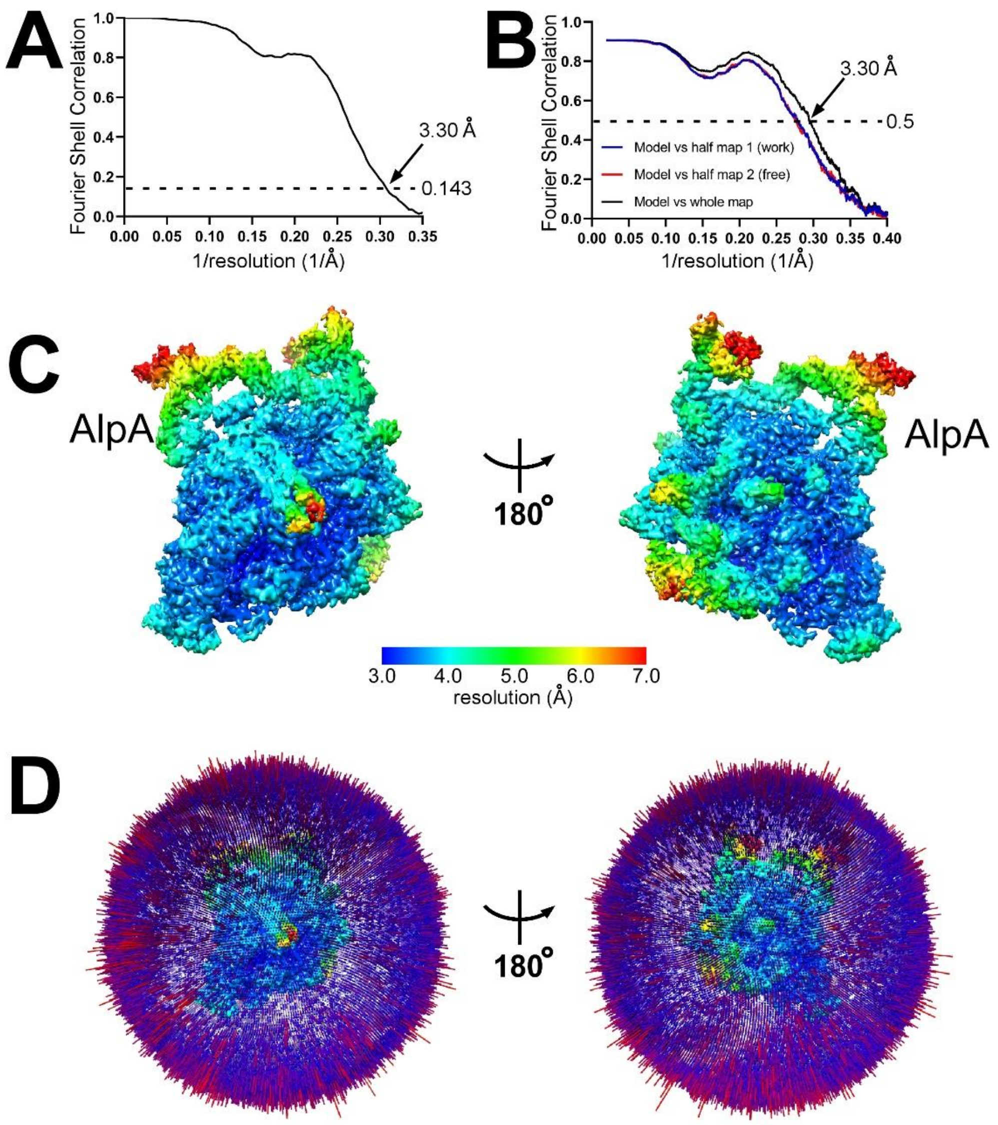

Figure S3. Data validation for AlpA-loading complex.

(A) Gold-standard FSC. The gold-standard FSC was calculated by comparing the two independently determined half-maps from RELION. The dashed line represents the 0.143 FSC cutoff.

(B) FSC calculated between the model and the half map used for refinement (work), the other half map (free), and the full map.

(C) Cryo-EM density map colored by local resolution. View orientations as in Figure 2B.

(D) Angular distribution of particle projections. View orientations as in Figure 2B.

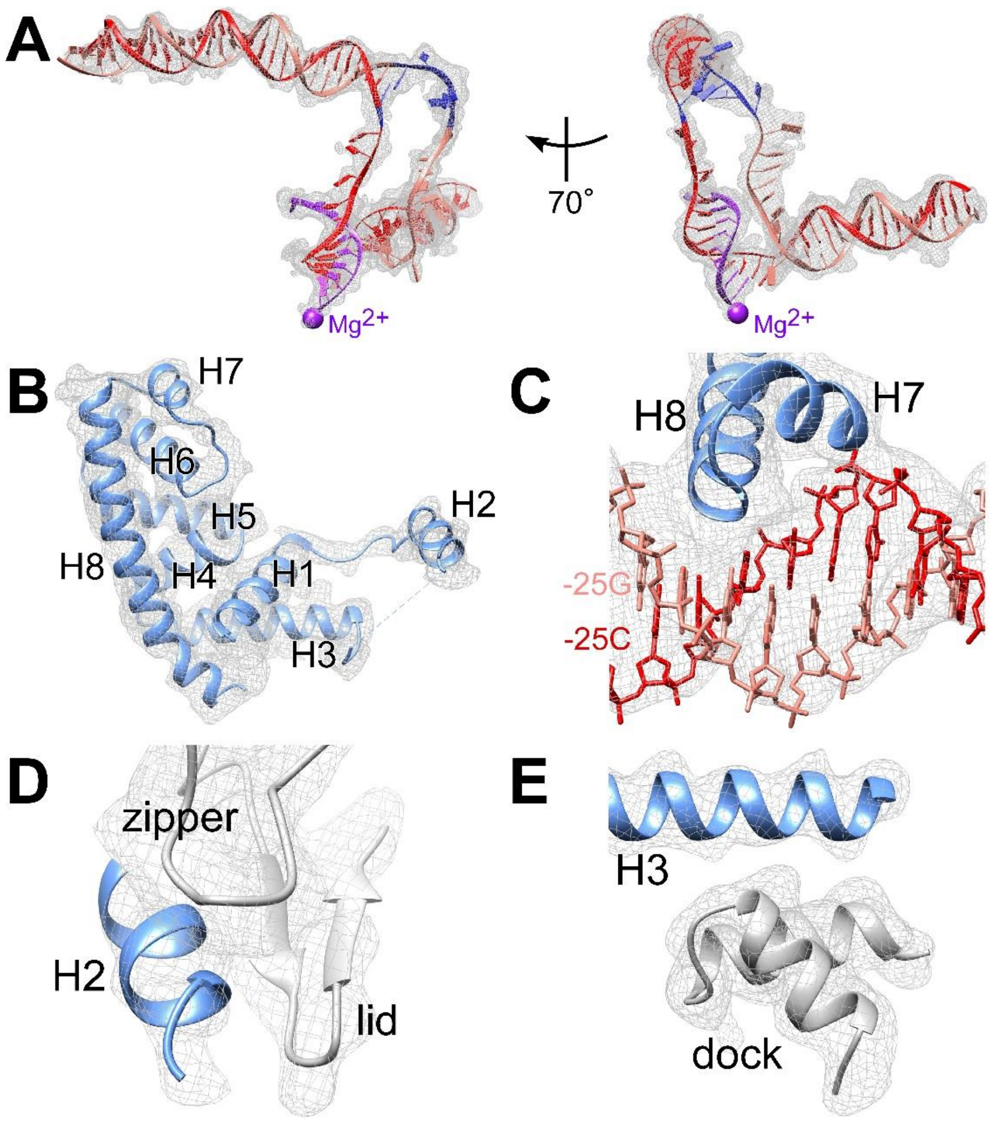

Figure S4. Representative cryo-EM densities and superimposed models of AlpA-loading complex.

(A) Cryo-EM density map without B-factor sharpening (gray mesh) and the superimposed model of the nucleic-acid scaffold.

(B) Cryo-EM density map without B-factor sharpening (gray mesh) and the superimposed model of AlpA.

(C) Cryo-EM density map without B-factor sharpening (gray mesh) and the superimposed model of H7, H8, and ABE.

(D) Cryo-EM density map without B-factor sharpening (gray mesh) and the superimposed model of H2, the zipper, and the lid.

(E) Cryo-EM density map without B-factor sharpening (gray mesh) and the superimposed model of H3 and the dock.

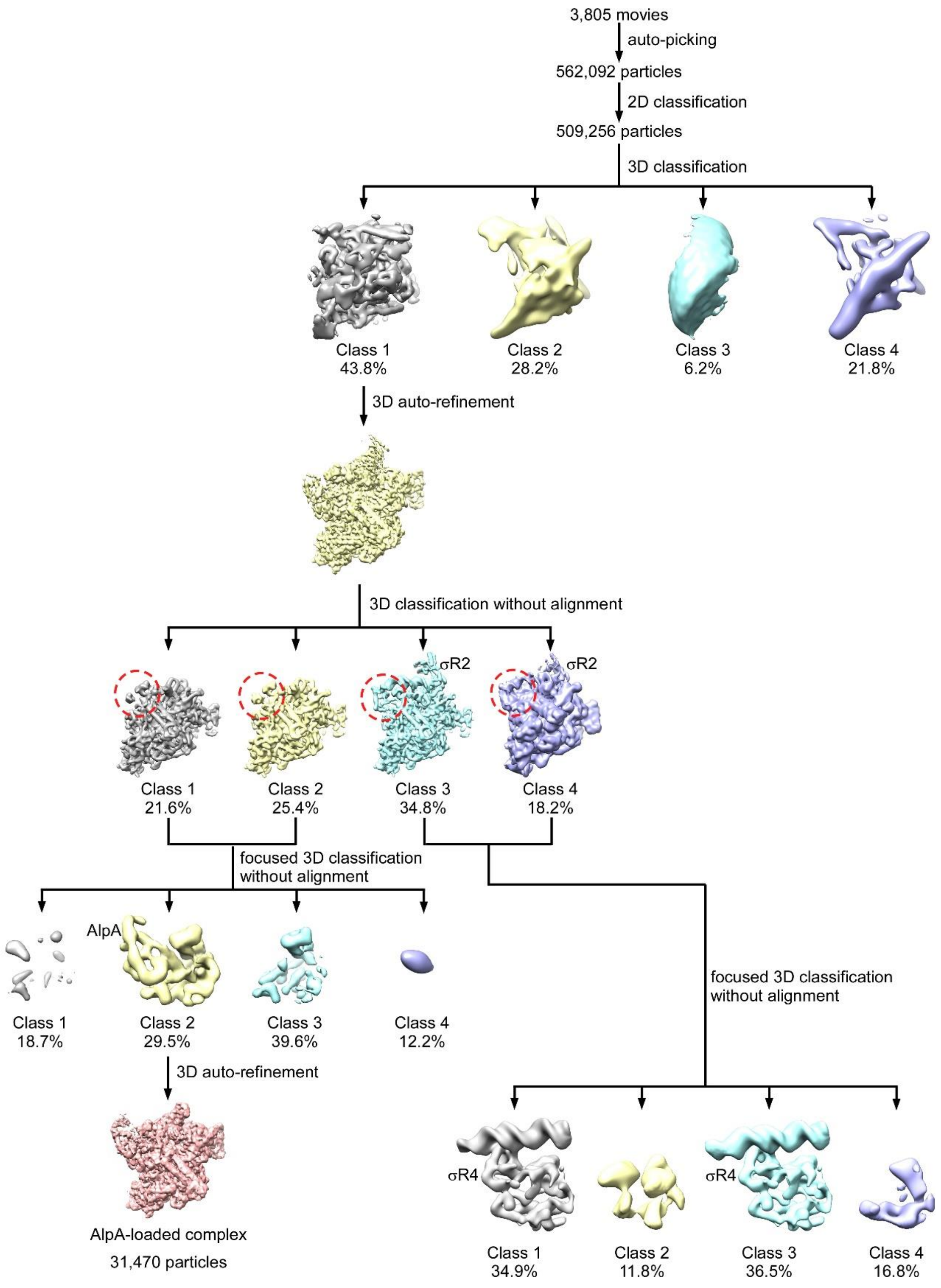

Figure S5. Data processing pipeline for AlpA-loaded complex.

3D classification resulted in 4 classes, among which only one class has a clear density for RNAP. Further 3D classification results in two classes with clear density for σ^{70} and two classes without density for σ^{70}. Particles without density for σ^{70} were combined, then subjected to 3D classification focused on the RNA exit channel without alignment. Focused 3D classification

resulted in 4 classes, among which only class 2 has a clear density for AlpA. Particles with density for σ^{70} were also subjected to 3D classification focused on the RNA exit channel without alignment. Focused 3D classification resulted in 4 classes, among which class 1 and class 3 have a clear density for σR4. The other two classes, which don't have any feature of α-helices, probably represent junk particles. Taking together, there is no complex with both AlpA and σ^{70} in this dataset, indicating that σ^{70} is released upon the formation of a loaded complex.

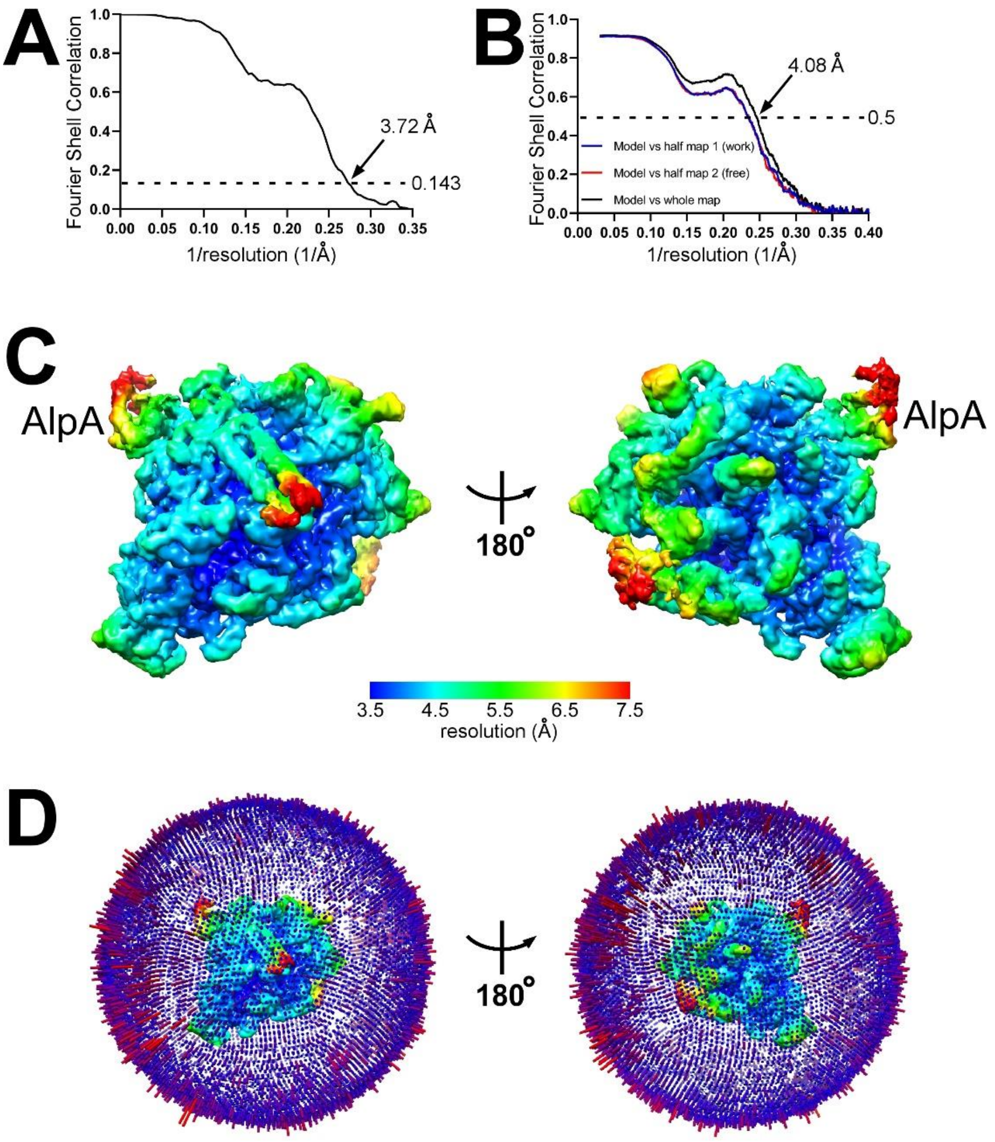

Figure S6. Data validation for AlpA-loaded complex.

(A) Gold-standard FSC. The gold-standard FSC was calculated by comparing the two independently determined half-maps from RELION. The dashed line represents the 0.143 FSC cutoff.

(B) FSC calculated between the model and the half map used for refinement (work), the other half map (free), and the full map.

(C) Cryo-EM density map colored by local resolution. View orientations as in Figure 5B.

(D) Angular distribution of particle projections. View orientations as in Figure 5B.

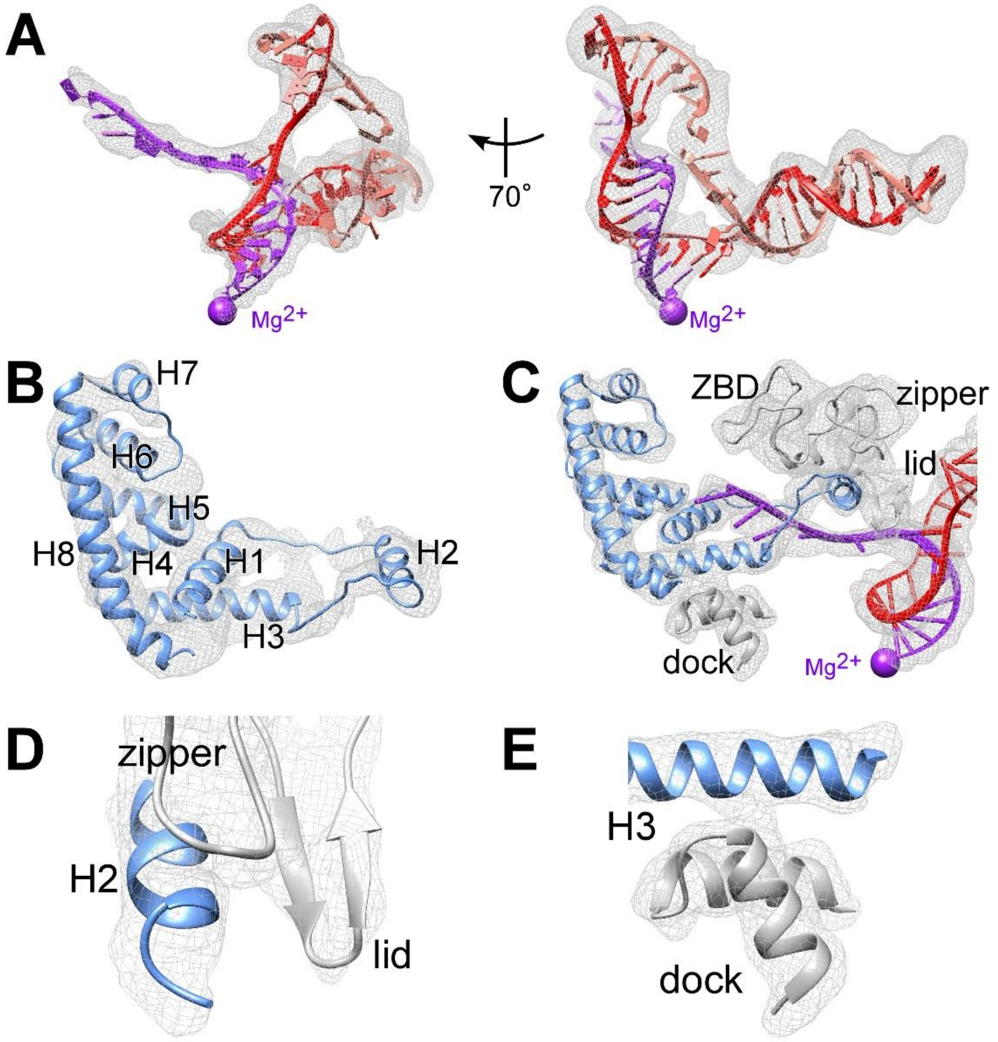

Figure S7. Representative cryo-EM densities and superimposed models of AlpA-loaded complex.

(A) Cryo-EM density map without B-factor sharpening (gray mesh) and the superimposed model of the nucleic-acid scaffold.

(B) Cryo-EM density map without B-factor sharpening (gray mesh) and the superimposed model of AlpA.

(C) Cryo-EM density map without B-factor sharpening (gray mesh) and the superimposed model of the RNA exit channel.

(D) Cryo-EM density map without B-factor sharpening (gray mesh) and the superimposed model of H2, the zipper, and the lid.

(E) Cryo-EM density map without B-factor sharpening (gray mesh) and the superimposed model of H3 and the dock.

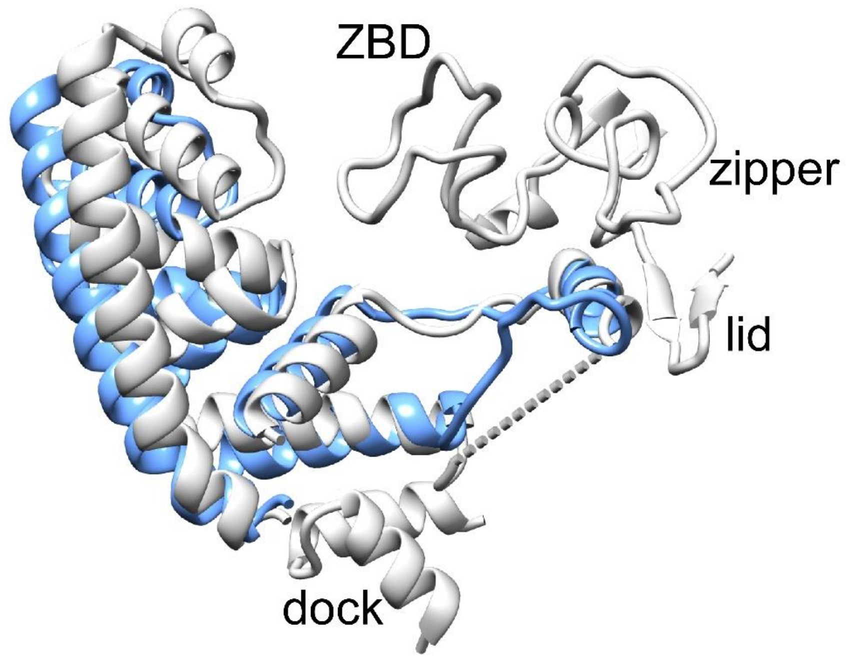

Figure S8. Superposition of AlpA-loading complex (gray) and AlpA-loaded complex (light blue).

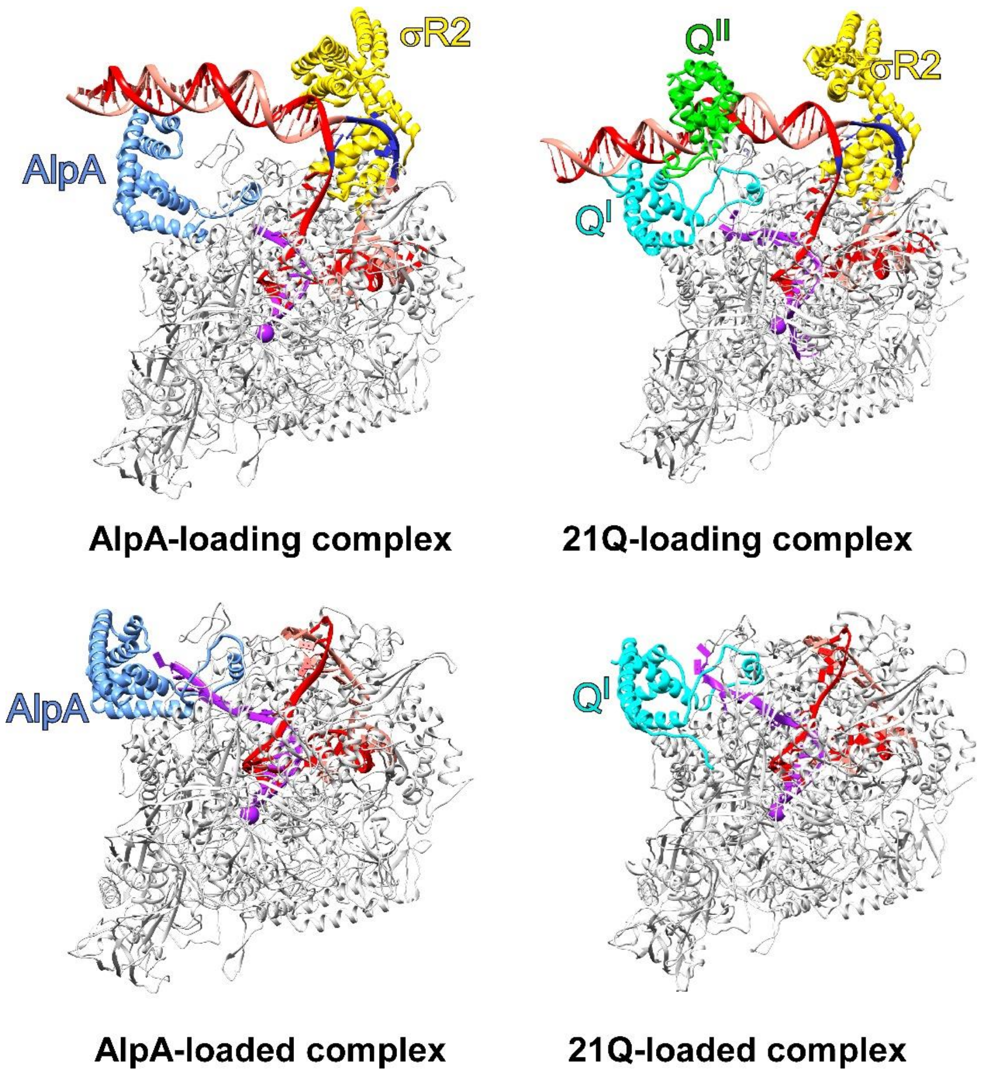

Figure S9. Structural comparison of AlpA-loading complex, 21Q-loading complex (PDB 6JNX), AlpA-loaded complex, and 21Q-loaded complex (PDB 6P19).

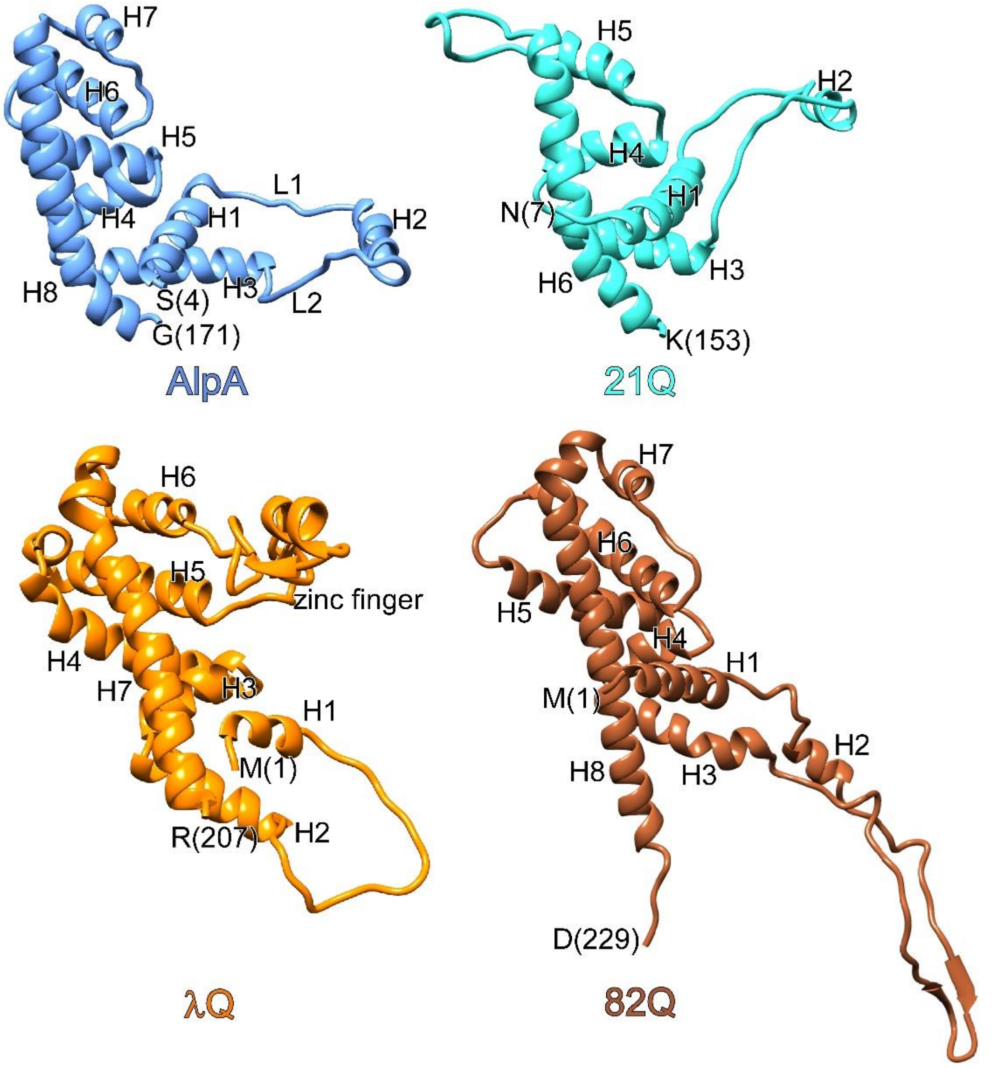

Figure S10. Structural comparison of AlpA, 21Q (PDB 6JNX), λQ (AlphaFold), and 82Q (AlphaFold).

Table S1. Cryo-EM data collection and refinement statistics.

	loading complex	loaded complex
**Data collection and processing**		
Microscope	Titan Krios	Titan Krios
Voltage (kv)	300	300
Detector	Falcon 4	Falcon 4
Electron exposure (e/Å^2)	62	52
Defocus range (μm)	1.0-2.0	0.9-1.8
Data collection mode	Counting	Counting
Physical pixel size (Å/pixel)	0.93	0.93
Symmetry imposed	C1	C1
Initial particle images	980,554	562,092
Final particle images	64,002	31,470
Map resolution (Å)[a]	3.3	3.7
**Refinement**		
Root-mean-square deviation		
Bond lengths (Å)	0.004	0.004
Bond angles (°)	0.655	0.726
Molprobity statistics		
Clashscore	11	17
Rotamer outliers (%)	0.7	0.6
Cβ outliers (%)	0	0
Ramachandran plot		
Favored (%)	96	95
Outliers (%)	0.06	0.06

[a]Gold-standard FSC 0.143 cutoff criteria.
